# Synthesis of Waterborne Polyurethane by the Telechelic α,ω-Di(hydroxy)poly(*n*-butyl acrylate)

**DOI:** 10.3390/polym10020219

**Published:** 2018-02-23

**Authors:** Xin Chen, Chi Zhang, Weidong Li, Lei Chen, Wusheng Wang

**Affiliations:** School of Chemistry and Chemical Engineering, Anhui University, 111 Jiu long Road, Hefei 230601, China; chenxinxg@foxmail.com (X.C.); AHUniubb@163.com (C.Z.); lwd0203@163.com (W.L.); 18225511660@163.com (L.C.)

**Keywords:** poly(*n*-butyl acrylate), degenerative iodine transfer, free-radical polymerization, waterborne polyurethane

## Abstract

A key for the preparation of polyacrylate-based polyurethane is the synthesis of hydroxyl-terminated polyacrylate. To our knowledge, exactly one hydroxyl group of every polyacrylate chain has not been reported. The hydroxyl-terminated poly(butyl acrylate) (PBA) has been successfully synthesized by degenerative iodine transfer polymerization (DITP) of the *n*-butyl acrylate (*n*-BA) using 4,4′-azobis(4-cyano-1-pentanol) (ACPO) and diiodoxylene (DIX) as initiator and chain transfer agent, respectively, and subsequently substituted reaction of the iodine-terminated PBA with β-mercaptoethanol in alkaline condition. The latter reaction was highly efficient, and the terminal iodine at the end of polymer chains were almost quantitatively transformed to a hydroxyl group. 2,2′-Azobis(isobutyronitrile) (AIBN) and ACPO were used as initiators in the DITPs of *n*-BA. The results demonstrated that they had a significant influence on the terminal groups of the formed polymer chains. The structure, molecular weight, and molecular weight distribution of the hydroxyl-terminated PBA have been studied by ^1^H, ^13^C NMR, and GPC results. The components of hydroxyl-terminated PBA were determined by MALDI-TOF MS spectra, and their formation is discussed. The broad molecular weight distribution of the PBA and the difference in the polymerization behaviors from typical living radical polymerization are explained based on the results of ^1^H NMR and MALDI-TOF MS spectra. The hydroxyl-terminated PBA has been successfully used in the preparation of PBA-based polyurethane dispersions (PUDs). The aqueous PUDs were stable, and based on the DSC results it can be said that the miscibility of hard segments with PBA chains was improved.

## 1. Introduction

Polyurethane dispersions (PUDs) are generally synthesized by the condensation polymerization of oligomer polyols, diisocyanate, chain extender, and hydrophilic comonomers. The oligomer polyols used in the preparation of PUDs are mostly di-functional oligomer diols, such as polyether diols, polyester diols, and polycarbonate diols [1]. Compared to these oligomers, the advantages of polyacrylate are good adhesion to the matrices, excellent light stability, and chemical resistance. PUDs are well-known for their peculiar advantages, such as high impact strength at low temperatures, tear resistance, and good elasticity [2,3,4,5]. If polyurethane and polyacrylate are combined in one polymer to form polyacrylate polyurethane, the newly formed PUDs may have the advantages of both polymers, which may have potential applications [6,7,8]. At present, the commercially available polyacrylate polyols have been manufactured by the copolymerization of acrylate monomers and hydroxy-alkyl acrylate monomers (e.g., 2-hydroxyethyl acrylate), and the hydroxyl groups are randomly distributed in the polymer side chains. In general, the hydroxyl content is 2–4%, and the average functionality is greater than three [9,10,11]. Because the structure and functionality of the polyacrylate polyol chains are inhomogeneous, and cross-linking reaction may occur when this kind of polyol reacts with isocyanate, polyacrylate polyols are seldom used to prepare aqueous PUDs. Although preparation of the waterborne polyurethane–polyacrylate hybrid emulsions has been reported, low compatibility between the polyurethane and the polyacrylate has limited its applications [12,13,14,15,16]. This problem can be solved by using the polyacrylate as soft segments of aqueous PUDs. The key issue for achieving this purpose is the synthesis of the oligomer polyacrylate diol. However, several review papers merely discuss the synthesis of the polyacrylate diols [17,18]; only one U.S. patent we were able to find in the literature involved the synthesis of hydroxyl-terminated polyacrylates [18]. It is difficult to believe that both ends of every polymer chain are capped with a hydroxyl group because 2,2′-azobis(isobutyronitrile) (AIBN) is used in the preparation, which will be discussed further.

Living radical polymerization (LRP) has been shown to be a viable method for the rational design of polymers with predictable molecular structure and narrow molecular weight distributions [19,20], and fundamental kinetic features of the LRP has been discussed [19,20]. Among several strategies of LRPs, degenerative iodine transfer polymerization (DITP) has been receiving our special attention because the iodine group at the end of the formed polymer can be almost quantitatively transformed to a hydroxyl group via a simple procedure. However, other LRPs (e.g., atom transfer radical polymerization and reversible addition-fragmentation chain transfer polymerization) can also be used in the preparation of polyacrylate diols. DITP controls chain growth through a reversible reaction of the growing chain radicals with iodo compound, limiting irreversible termination of the growing chain radicals; the mechanism of DITP and DITP in the presence of halogenated monomers have been discussed in detail [21]. A number of homopolymers and block copolymers including polystyrene (PS), poly(methyl methacrylate) (PMMA), and poly(vinyl chloride) (PVC) have been successfully synthesized through DITP [20,22,23,24,25,26]. In the case of solution polymerization, a large variety of solvents (e.g., toluene, anisole, etc.) can be used as polymerization media [20,22]. DITP has been successfully used in emulsion polymerization for the preparation of polystyrene-*b*-poly(butyl acrylate) [20,22,27]. Many studies have demonstrated that the DITP is a feasible method for the synthesis of low-molecular-weight polyacrylate (*M*_n_ = 2000–6000 g mol^−^^1^), but the ratio of iodine-end-capped functional groups cannot reach 100% (mostly only 50–80%). Besides the iodine, the other end group comes from the initiator fragment [28,29]. Conversion of the end-iodine groups into hydroxyl group has been studied [18,30], but the yielded product cannot be used in synthesis of high molecular weight polyurethane.

The purpose of the study reported herein is to synthesize a well-defined α,ω-di(hydroxy)poly(*n*-butyl acrylate) with relatively low molecular weight, and then to use it in the synthesis of PUDs with polyacrylate as soft segment.

## 2. Materials and Methods

### 2.1. Materials

4,4′-Azobis(4-cyano-1-pentanol) (ACPO) was purchased from Henan Lienchem Inc. (Zhengzhou China). 2,2′-Azobis(isobutyronitrile) (AIBN) was bought from Energy Chemical (Shanghai, China) and purified by recrystallization in ethanol. *n*-Butylacrylate (*n*-BA), α,α′-dibromo-*p*-xylene, decane, d-chloroform (CDCl_3_), and isophorone diisocyanate (IPDI) were purchased from Macklin (Shanghai, China). Toluene, 2-mercaptoethanol, 2,2-dimethylol propionic acid (DMPA), and acetone were purchased from Sinopharm Chemical Reagent Co., Ltd. (Shanghai, China). Calcium oxide, potassium carbonate (K_2_CO_3_), 1,4-butanediol, and triethylamine were bought from Aladdin (Shanghai, China). All other chemicals were used as received.

### 2.2. Methods

^1^H nuclear magnetic resonance (^1^H NMR) and ^13^C nuclear magnetic resonance (^13^C NMR) (400 MHz) were performed at room temperature on AVANCEII 400 MHz spectrometers in CDCl_3_ using tetramethylsilane as an internal standard.

Molecular weight and molecular weight distribution were measured on a PL-GPC120 gel permeation chromatograph (GPC) equipped with a PLGel 5 μm column (300 × 7.5 mm purchased from Polymer Laboratories (Santa Clara, CA, USA)) and a refractive index detector (G1362A 1100/1200 series refractive index detector (Agilent Technologies Inc., Santa Clara, CA, USA) at 40 °C. Tetrahydrofuran (THF) was used as solvent at a flow rate of 1 mL/min. The monodisperse polystyrene standards (test range of molecular weights was from 1000 to 200,000 g/mol, Polymer Laboratories) were utilized in the calibration of *M*_n_, *M*_w_, and *M*_w_/*M*_n_. Sample concentration was 1 mg/mL, and sample injection volume was 300 μL.

Mass-assisted laser desorption ionization time-of-flight mass spectrometry (MALDI-TOF) measurements were performed on a Bruker Autoflex III TOF/TOF mass spectrometer (Bruker Daltonics Inc., Billerica, MA, USA). The instrument was operated in positive ion reflection mode with an accelerating potential of +20 kV. *trans*-2-[3-(4-*tert*-butylphenyl)-2-methyl-2-proprnylidene] malononitrile was used as a matrix and dichloromethane as a solvent. Sodium iodide was dissolved in methanol and used as the ionizing agent. Samples were prepared by mixing 2 μL of polymer solution with 2 μL of matrix solution. Then, 1.0 μL of these mixtures was deposited on a target plate and the solvent was removed in a stream of nitrogen.

The hydroxyl number of the α,ω-di(hydroxy)poly(*n*-butyl acrylate) was determined by the phthalic anhydride reflux method in accordance with ASTM Standard D 2849.

Differential scanning calorimetry (DSC) analysis was conducted on a Q2000 of TA instruments (TA Instruments, New Castle, DE, USA) in the range of −80 °C to 150 °C. The experiments were carried at a heating rate of 20 °C/min under nitrogen flow at a rate of 20 mL/min. The samples were dried at 65 °C for 48 h under vacuum prior to analysis.

The stability of emulsions was characterized by centrifugation at 3000 rpm/min for 15 min at room temperature, and then were observed to evaluate whether precipitation occurred.

Particle size and particle size distribution were measured at 25 °C with Zetasizer Nano series ZS 90 (Malvern Instruments Ltd., Malvern, UK). The samples were diluted to 1.85% (mass concentration) in distilled water.

### 2.3. Synthesis of Diiodoxylene

The synthetic procedure was similar to the previous report [23,31]. Sodium iodide (0.08 mol) and α,α′-dibromo-*p*-xylene (0.03 mol) were dissolved in acetone under nitrogen. The reaction was carried out at room temperature, and after subsidence, the stirring was continued for an additional 30 min. The product was precipitated by adding water (250 mL), and the salt formed was dissolved in water. Then, the precipitate was removed by filtration, washed with water five times, and the final product was obtained by drying in a vacuum oven at room temperature overnight.

### 2.4. Degenerative Iodine Transfer Polymerization of n-BA

*n*-BA (0.39 mol), ACPO (0.02 mol), diiodoxylene (0.04 mol), and toluene (120 mL) were placed in a 500 mL round-bottom flask protected in the dark. The reaction mixture was heated in a nitrogen atmosphere at 60 °C under magnetic stirring. After 24 h, the polymerization solution was cooled in an ice bath. Subsequently, potassium carbonate (K_2_CO_3_) was added into the flask in order to change the pH of the reaction mixture to alkalinity, and then β-mercaptoethanol was added to the reaction mixture for transformation of end-iodine to end-hydroxyl group. The reaction solution was collected by filtration through 1.0 μm microporous filters, and toluene in the collected solution was removed by rotary evaporation. Then, the target product was purified by washing with ethanol and distilled water. After the ethanol in the product was removed in a vacuum oven, a sample dissolved in toluene was heated at 110 °C for the removal of water and the purified product was obtained.

### 2.5. Kinetics Study of Poly(n-butyl acrylate) by Degenerative Iodine Transfer Polymerization

In order to study the kinetic features of this bulk polymerization system, we carried out the polymerizations at 60 °C and took samples periodically at the designed polymerization time (24 h). Monomer conversion was determined by gravimetric analysis. Several drops of 4-hydroxyanisole (MEHQ) (0.5 wt %) were added to 0.5 mL of polymer solution every 30 min. Then, we placed the sample in an ice bath. The small molecules in solution were then removed by vacuum drying at 65 °C. The weight before and after drying was compared, and monomer conversion was obtained by the equation:

$$\mathrm{Conversion}\;\left(\%\right)=\;\frac{m_{3}}{m_{1}\;-\;m_{2}}\times 100\%$$

where *m*_1_ is the weight of solution, *m*_2_ is the weight of solvent, and *m*_3_ is the weight of dried polymer. The obtained polymer was characterized by ^1^H NMR spectroscopy.

### 2.6. Synthesis of Polyacrylate-Based Polyurethane Dispersions (PUDs)

Poly(*n*-butyl acrylate) diol (6.67 × 10^−3^ mol) was charged under stirring, and the isophorone diisocyanate (IPDI) (0.03 mol) was then slowly added into the diol. The mixture was then heated to 80–85 °C until the theoretical isocyanate group (NCO) content of the prepolymer was reached, which was estimated using the di-*n*-butylamine titration method. Then, the chain extenders 1,4-butanediol and dimethylolpropionic acid (DMPA) (9.40 × 10^−3^ mol) were added into the mixture at the same temperature for 1.0 h through adjusting the viscosity by acetone. Distilled water (106 mL) was then added into the neutralized PU by using triethylamine (9.4 × 10^−3^ mol) as a neutralizing agent after adding catalyst. An aqueous dispersion of 20 wt % solids was obtained after removal of acetone by decompressing distillation.

## 3. Results and Discussion

### 3.1. Design of DITP System for Preparation of Hydroxyl-Terminated Poly(butyl acrylate) 

**Figure 1:**
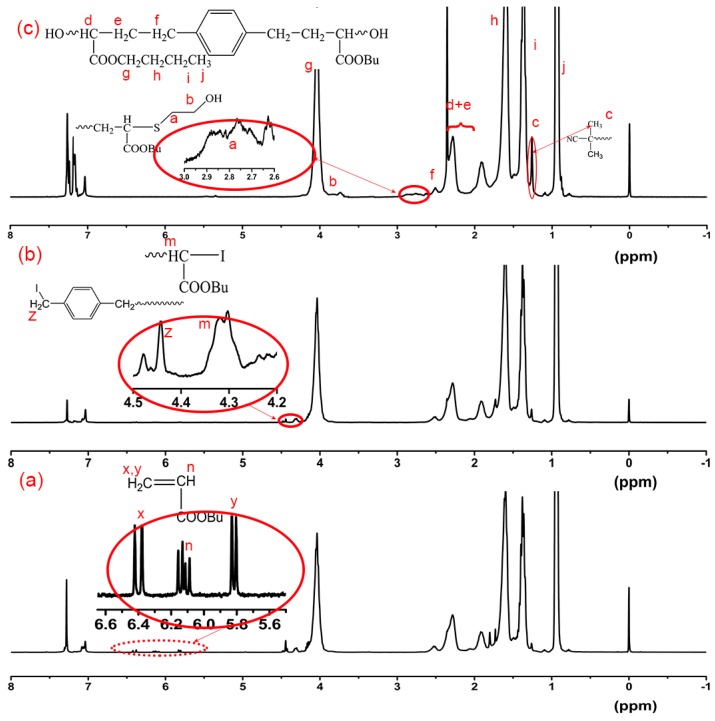
^1^H NMR spectra of the polymers respectively obtained from (**a**) the DITP of *n*-BA using AIBN as initiator and DIX as transfer agent for 1 h, and (**b**) for complete consumption of BA. (**c**) The polymer obtained after converting the iodide group at the ends of the polymer in (b) into the hydroxyl group through reaction with β-mercaptoethanol.

To prepare low-molecular-weight poly(butyl acrylate) (PBA) with both ends capped with hydroxyl groups via DITP, the first issue is the design of an appropriate polymerization system. According to previous reports [18,32], hydroxyl-terminated polyacrylate could be prepared by the DITP of acrylates using diiodoxylene (DIX) and AIBN as di-functional chain transfer agent and initiator, respectively, and this patent claimed that the end-functionalized polymers were useful as reactive intermediates in condensation polymerization, chain polymerization, and heterogeneous polymerization. Therefore, we first prepared the PBA diols according to the reported method [18]. The results are listed in Table 1, and the molecular weight was determined by GPC, the OH value was measured by phthalic anhydride reflux method, and the functionality of end-hydroxyl group was calculated based on ^1^H NMR spectra as shown in Figure 1. Surprisingly, we observed that the functionality of end-hydroxyl group in the resultant polymers was less than 60% (Table 1). Thus, it is necessary to clarify what happened in this polymerization.

**Scheme 1:**
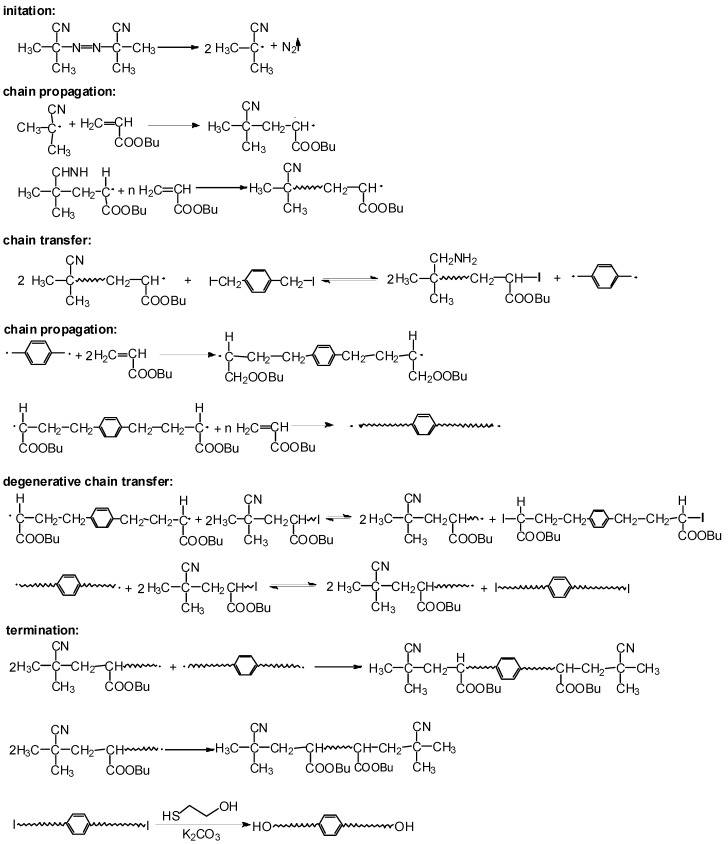
Mechanism of the degenerative iodine transfer polymerization of *n*-BA.

Based on the mechanism of DITP reported in References [21,24], the DIPT mechanism of BA can be depicted in Scheme 1. The chain growth is controlled by reversible reaction of the growing chain radicals with DIX, and after the polymerization, the xylene groups are located in the middle of PBA chain; however, the PBA without xylene group cannot be excluded. According to this mechanism, the chain end groups—which are of especial interest in this study—include iodine and isobutyronitrile groups. When the β-mercaptoethanol was added into the reaction system, the iodine at the end of the formed polymer chains could be converted to a hydroxyl group. In order to verify these results, ^1^H NMR was used to follow the polymerization, and typical ^1^H NMR spectra are shown in Figure 1.

Figure 1a,b show the proton NMR spectra of the PBA obtained respectively from DITP for 1 h and complete conversion of *n*-BA. The proton signals at δ = 4.04, 2.36, and 1.91 ppm are respectively ascribed to the ester methylene group and the methine and methylene groups in the backbone of the PBA chain; the aromatic protons and two methylene protons of the xylene units appear at δ = 7.05 and 2.51 ppm, respectively. Other proton signals of the PBA are marked in Figure 1, and the vinyl signals of *n*-BA monomer at 6.38, 6.14, and 5.83 ppm completely disappear in Figure 1b; all of these results indicate that PBA was successfully synthesized. The signal of methylene protons in the DIX unit at δ = 4.45 ppm almost disappeared, and a new signal at δ = 4.30 ppm appeared in Figure 1a,b, which is attributed to the methine proton of the BA unit next to terminal iodine. This demonstrates that reversible transfer reaction of the growing chain radical with DIX occurred, forming *p*-iodomethylene benzyl radicals and then initiating *n*-BA to polymerize, producing PBA. Based on the integration ratio of the signal at 4.30 ppm and the signal at 4.45 ppm, approximately 90% of the DIX units were located in the middle of PBA chains.

When the iodine-terminated PBA was treated with β-mercaptoethanol, the hydroxyl-terminated PBA was obtained, and its proton NMR spectrum is shown in Figure 1c. We can see that the proton signals at δ = 4.45 and 4.30 ppm completely disappeared, two new proton signals appeared in Figure 1c: one signal at δ = 3.73 ppm, which is ascribed to methylene protons next to the terminal hydroxyl group; another at δ = 2.67–2.96 ppm, which is attributed to methylene protons adjacent to an ether sulfur atom. These results demonstrate that the substitute reaction of HOCH_2_CH_2_SK with PBA-I is almost quantitative. When we carefully analyze the ^1^H NMR spectrum in Figure 1c, we can observe a proton signal at δ = 1.26 ppm, which is ascribed to methyl protons of the isobutyrontrile unit. Based on the polymerization mechanism shown in Scheme 1, this initiator fragment should stand at the end of the PBA chain. Therefore, the obtained PBA has two end groups: hydroxyl and isobutyrontrile. The hydroxyl functionality of the obtained PBA can be calculated based on integral values of the signals at δ = 1.26 and 3.73 ppm, and the results are listed in Table 1. Theoretically, there are three types of PBA based on their different end groups, HO–PBA–OH, HO–PBA–I, and I–PBA–I (I refers to isobutyrontrile group). The results in Table 1 show that the hydroxyl functionality of all obtained PBAs is approximately 50%; the molar percentage of HO–PBA–OH—which is expected in the preparation of aqueous PUDs—was less than 50%. Such hydroxyl-functionalized PBA will significantly influence the properties of the formed aqueous PUDs because the I–PBA–I is inert in the reaction with diisocynates and the HO–PBA–I terminates the condensation polymerization of diol with diisocynate monomers. To synthesize the PBA with 100% hydroxyl functionality, the design of a new formulation is necessary. Based on the above discussion, the low hydroxyl functionality of the PBA is due to the use of AIBN without hydroxyl group as initiator; thus, the initiator ACPO with two terminal hydroxyl groups was selected and used in the DITP of *n*-BA.

### 3.2. DITP of n-BA Using DIX and ACPO as Transfer Agent and Initiator

**Figure 2:**
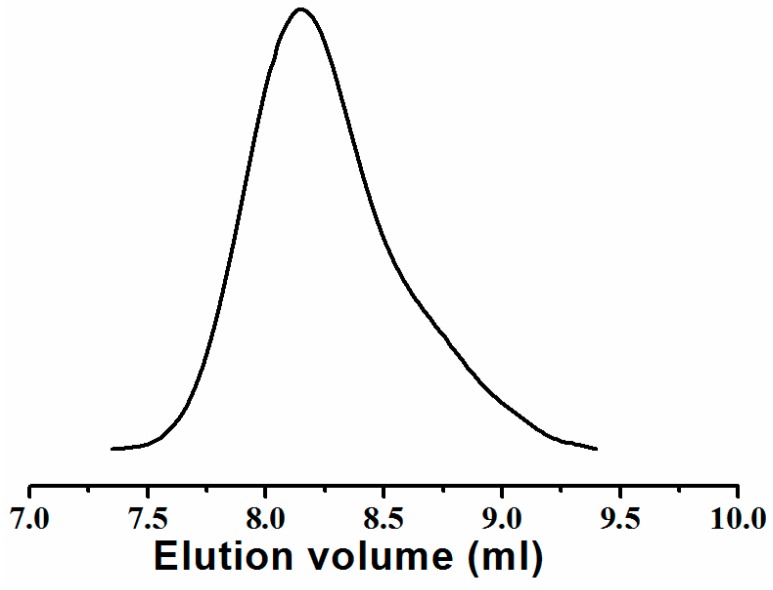
Gel permeation chromatography (GPC) curves of the polymer obtained from the DITP of *n*-BA with feed molar ratios of [*n*-BA]/[ACPO]/DIX] = 16/0.08/1 (*M*_n,GPC_ = 3200 g/mol).

In order to synthesize low-molecular-weight PBA with 100% hydroxyl functionality for application in the preparation of aqueous PUDs, low feed molar ratio of monomer to transfer agent is generally required. Thus, the DITP of *n*-BA with feed molar ratio of [*n*-BA]/[ACPO]/[DIX] = 16:0.08:1 was conducted at 60 °C for 24 h. The PBA was obtained by precipitation, filtration, and drying, and its GPC curve and ^1^H NMR spectrum were measured. Figure 2 shows its GPC curve, which reveals a single curve with a pronounced tail. The probable reason is irreversible termination of the growing chain radicals, because at the initial stage of polymerization, fast decomposition of initiator ACPO produced too many primary radicals, and then the growing chain radicals could not be efficiently captured by transfer agent (DIX), leading to their irreversible termination. The tail of the GPC curve led to a broad molecular weight distribution (*M*_w_/*M*_n_ = 1.67).

**Figure 3:**
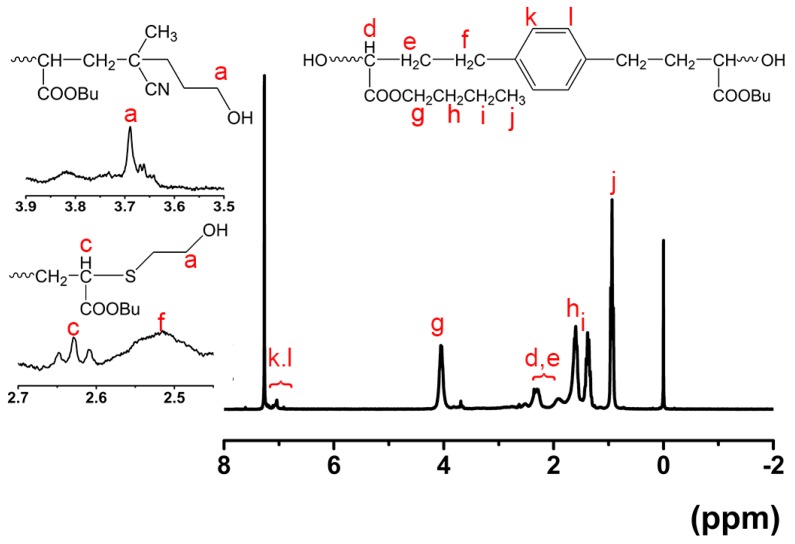
^1^H NMR spectrum of the poly(butyl acrylate) (PBA) synthesized by DITP of the *n*-BA with feed molar ratio of [*n*-BA]/[ACPO]/[DIX] = 16/0.08/1 at 60 °C for 24 h (*M*_n,GPC_ = 3200 g/mol). ACPO: 4,4′-azobis(4-cyano-1-pentanol).

After the iodine-terminated PBA was treated with β-mercaptoethanol in alkaline conditions, the hydroxyl-terminated PBA was obtained, and its ^1^H NMR spectrum was measured and is shown in Figure 3. Similar to the ^1^H NMR spectrum of PBA obtained by initiation of AIBN, the characteristic ester methylene proton signal, the methane, and methylene proton signals in the PBA backbone appear at δ = 4.08, 2.33, and 1.96 ppm, respectively, demonstrating the successful synthesis of PBA. The iodomethylene proton signal of DIX unit at δ = 4.45 ppm completely disappeared, and a new proton signal clearly appeared at δ = 2.57 ppm. This is because the reversible reaction of the growing PBA chain radicals with DIX produced *p*-iodomethylene benzyl radicals and the iodine-terminated PBA, and the formal radicals initiated the polymerization of *n*-BA to form PhCH_2_–*n*-BA linkage. So, the methylene proton signal of the DIX was shifted from δ = 4.45 to 2.57 ppm. As in the discussion of the ^1^H NMR spectra in Figure 1, the end groups of PBA come from initiator and transfer agent. The terminal iodine of PBA–I chains was converted to a hydroxyl group by reaction of PBA–I with HO–CH_2_CH_2_SK, which is confirmed by its ^1^H NMR spectrum in Figure 3. The signal of methine proton in the terminal *n*-BA unit at δ = 4.30 ppm completely disappeared, and two proton signals at δ = 2.67 and 3.62–3.93 ppm appeared in Figure 3, which are respectively attributed to two methylenes, respectively adjacent to sulfur and hydoxyl groups. The proton signal of the terminal hydroxyl group, which comes from the ACPO initiator, also appears at δ = 3.62–3.93 ppm. Because the proton number of the two methylene and the phenylene in the DIX is the same, the contribution ratio of the initiator ACPO and transfer agent DIX to the terminal hydroxyl group can be estimated based on the integration ratio of the signals at δ = 3.62–3.93 and 7.04 ppm, and is 0.07/1—close to the feed molar ratio of [ACPO]/[DIX] = 0.08/1.

**Figure 4:**
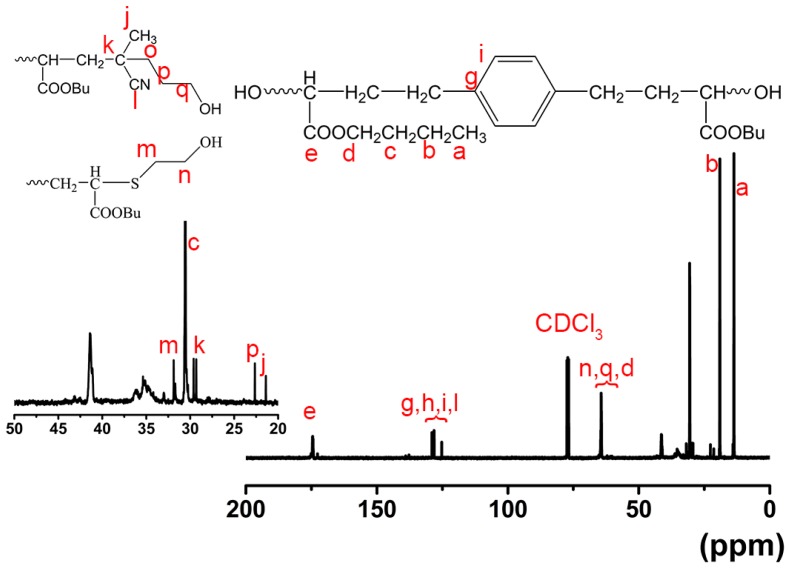
^13^C NMR spectrum of the PBA synthesized by DITP of the *n*-BA with feed molar ratio of [*n*-BA]/[ACPO]/[DIX] = 16/0.08/1 at 60 °C for 24 h (*M*_n,GPC_ = 3200 g/mol).

The ^13^C NMR spectrum in Figure 4 supports the structure of hydroxyl-terminated PBA, which was obtained based on analysis of its ^1^H NMR spectrum in Figure 3; the signal of the terminal hydroxylmethylene carbon appears at δ = 64.29 ppm, and the ascription of the other carbon signals in the hydroxyl-terminated PBA are marked in Figure 4.

**Figure 5:**
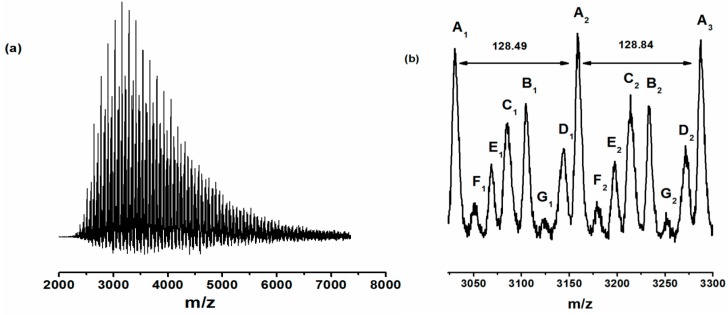
(**a**) Mass-assisted laser desorption ionization time-of-flight mass spectrometry (MALDI-TOF) spectrum and (**b**) enlarged spectrum of the hydroxyl-terminated PBA obtained by DITP of *n*-BA with feed molar ratio of [*n-*BA]/[ACPO]/DIX = 16/0.5/1 in toluene at 60 °C for 24 h (*M*_n,GPC_ = 4280 g/mol).

To further verify the structure and the terminal groups of PBA, its MALDI-TOF mass spectra were measured. MALDI-TOF mass spectrometry is a powerful technique for gaining mechanistic insights into polymerization reactions based on the presence or absence of mechanism-specific reaction products [33]. Figure 5a shows a typical MALDI-TOF mass spectrum of the hydroxyl-terminated PBA with *M*_n,GPC_ = 4280 g/mol, and its enlarged spectrum ranging from 3025 to 3300 g/mol is shown in Figure 5b. We can see seven series of the PBA chains that repeat with the mass of an *n*-BA unit (128 g/mol, see Figure 5b), and the structural formulas for each series of polymers—depicted based on their mass—are listed in Table 2. Among the seven series, series A is a relatively abundant PBA series. Based on the mass of series A, a structural formula can be depicted and is Na^+^HOCH_2_CH_2_CH_2_(CN)(CH_3_)C–(*n*-BA)_n_–SCH_2_CH_2_OH. When the degree of polymerization (DP) was 23, its number-average molecular weight (*M*_n_) could be calculated as 3159.2 g/mol, which is very close to the measured value (3158.4 g/mol).

Based on the careful analysis of the structural formulas of seven series of PBAs in Table 2, we can conjecture interesting reactions occurring in the polymerization and clarify how the terminal groups formed. As we mentioned previously, the end groups of PBA come only from the initiator, ACPO, and transfer agent, DIX. This can be further confirmed by the results of MALDI-TOF analysis. Both ends of the PBA capped with ACPO fragments include series D, E, and G; the transfer agent fragment, I—which was substituted by 2-mercaptoethanol after polymerization—occupies both ends of series B and F. Both end units of series A and C were ACPO and DIX fragments, respectively. Because the ACPO fragment has one terminal hydroxyl group, and after iodine at the ends of PBA was quantitatively substituted by KSCH_2_CH_2_OH (Figure 3), the PBA with hydroxyl groups at both ends was successfully synthesized, which is consistent with the results based on the analysis of ^1^H NMR spectrum.

**Scheme 2:**
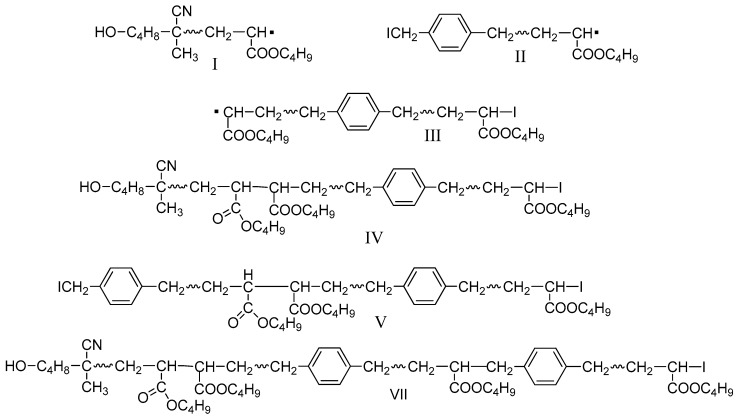
Possible intermediates formed in the DITP of *n*-BA.

The reactions occurring in the polymerization could be gained based on the presence or absence of mechanism-specific reaction products [33]. Based on the DITP mechanism listed in Scheme 1, we can depict how seven series of the PBA in Table 2 are formed; there are probably several routes to reach these PBA species, and herein we describe only one of them. The intermediates for the formation of final PBA species are depicted in Scheme 2. The primary radicals formed via decomposition of the ACPO initiates the polymerization of *n*-BA to yield PBA chain radical I, and then it reversibly reacts with DIX to form PBA–I and *p*-iodomethylene benzyl radical. The latter initiates *n*-BA to polymerize, yielding the PBA chain radical II. When the iodomethylene of radical II reacts with the growing chain radical or primary radical and then initiates the polymerization of *n*-BA, the growing chain radical III is formed. If the growing chain radical I continuously grows through chain propagation and degenerative chain transfer, series A is formed. Irreversible termination of radical I yields series E. Series B is formed through continuous growth of radical II, and then radical III is capped with iodine via its reaction with growing chain radicals. Coupling reaction of radical I with radical III produce the PBA species IV; after reaction with the growing chain radicals, the resultant radical IV is coupled to form series D. Termination reactions of radical III with radical II or radical IV, respectively, yielded PBA species V or VI. Radical V is terminated with radical III or with radical IV, respectively, to afford series F or series C, and series G is produced through coupling reaction of radical IV with radical VII. Therefore, the hydroxyl-terminated PBAs obtained by DITP of *n*-BA are a mixture composed of a series of PBA with different numbers of DIX units and terminal species. Indeed, the reactions in the DITP of *n*-BA are more complicated than that described above; all these reactions will influence the polymerization kinetics and the formation of polymer chains, which will be discussed later.

**Figure 6:**
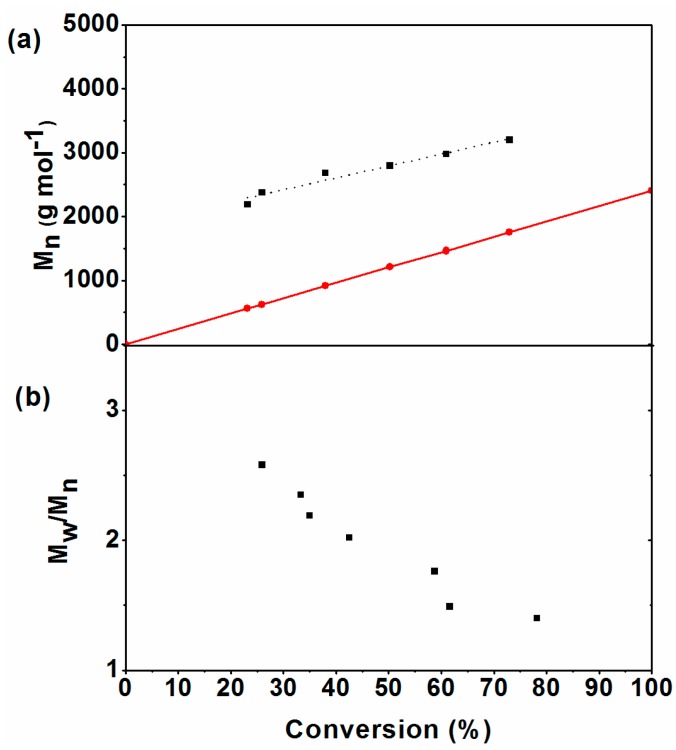
Degenerative iodine transfer polymerization of *n*-BA with molar ratio of [*n*-BA]/[ACPO]/[DIX] = 16/0.08/1 at 60 °C for different times. Dependence of (**a**) molecular weight and (**b**) molecular weight distribution on the conversion of *n*-BA. The line with solid circles presents the theoretical number-average molecular weight; and the line with solid squares is the molecular weight obtained by GPC in (a).

One purpose of this study was the synthesis of low-molecular-weight PBA; thus, the evolution of molecular weight versus monomer conversion was tested, and the results are shown in Figure 6. One point we emphasized is that *M*_n,GPC_ was obtained by GPC measurement. Although we can observe an almost linear increase of the *M*_n,GPC_ with *n*-BA conversion in Figure 6a, the difference of *M*_n,GPC_ with average-number theoretical molecular weight (*M*_n,theo_) decreased with increase of the *n*-BA conversion, and at high conversion (approximately 90%), the *M*_n,GPC_ approached *M*_n,theo_. This phenomenon has been reported by other researchers; their explanation is a low degenerative chain transfer constant [34]. Owing to this low constant, the primary radicals or the growing chain radicals cannot be rapidly reacted with DIX, and irreversible termination of the active radicals easily occurred to form dead polymer chains—series E in Table 2. Besides series E, series D and G are also dead polymer chains, which led to a decrease of the active radicals in the polymerization system. Thus, as reported in the study of kinetic modeling [35], the ratio of transfer agent/active radicals increased with the increase of monomer conversion; as a result, irreversible termination of the growing chain radicals significantly decreased. For the same reason, the molecular weight distributions (MWDs) of the resultant PBA became narrow with the increase of the *n*-BA conversion, but MWDs of all the PBA obtained were broader in comparison with the LRP, as shown in Figure 6b. Besides the irreversible termination of growing chain radicals, another probable reason is that the PBA chains have different numbers of active centers; series B and F have two initiator fragments, but series A and C have only one initiator fragment. The chain growth rates between series B (F) and series A (C) were different, which led to a broad molecular weight distribution.

In order to study the influence of feed molar ratio on the molecular weight of resultant PBA, the DITP of *n*-BA with various feed molar ratios of [ACPO]/[DIX] was studied, and the results are listed in Table 3. We can see that reducing the relative amount of the initiator, ACPO, did not obviously influence the molecular weight of the obtained PBA.

### 3.3. Synthesizing Aqueous Polyurethane Dispersions with α,ω-Di(hy-droxy)poly(n-butyl acrylate)

After the low-molecular-weight PBA with well-defined terminal group was successfully prepared, the important issue was to determine whether the resultant PBA could be used in the synthesis of aqueous polyurethane dispersions with PBA as soft segment. Aqueous PUDs are generally preferred in applications because they are environmentally friendly compared to the polyurethane solution. We defined this novel aqueous polyurethane dispersion as PBA-based PUD, which has not been reported in the literature, and may have some theoretical and industrial value.

**Figure 7:**
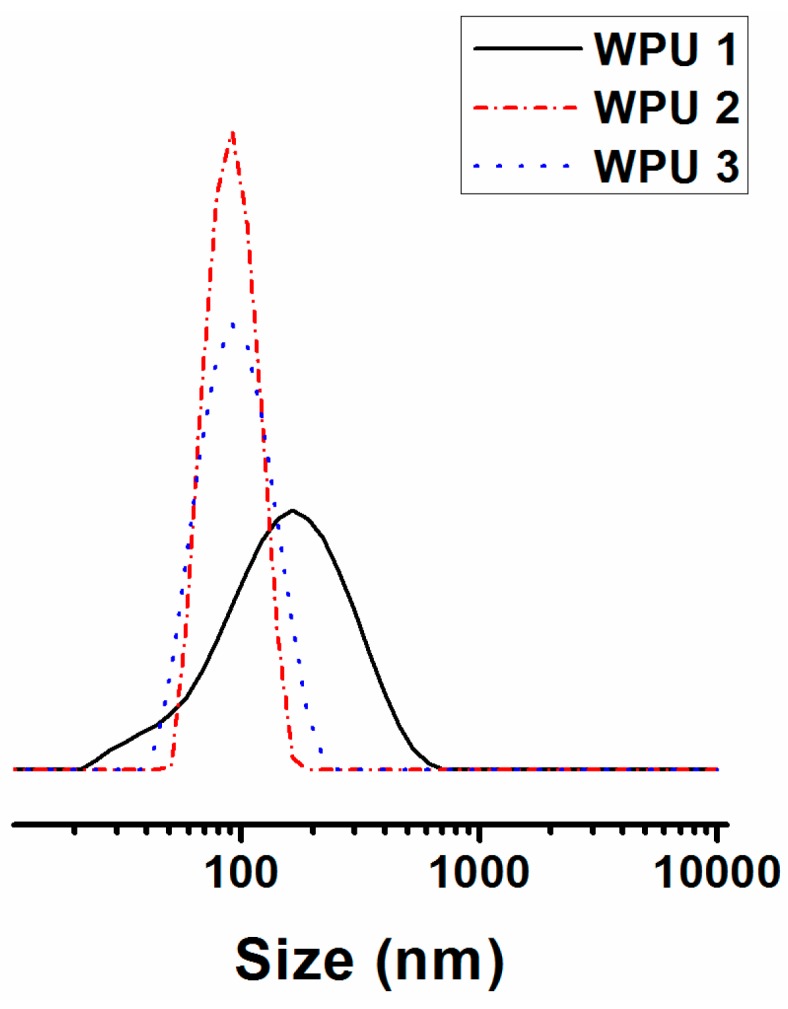
The particle size distribution diagram of the aqueous polyacrylate-based polyurethane by poly(*n*-butyl acrylate) diol.

The three hydroxyl-terminated PBA samples listed in Table 3 were respectively used in the synthesis of aqueous PUDs, and the obtained aqueous PUDs are respectively denoted as WPU_1_ to WPU_3_ (WPU_1_ was prepared from Exp. 1 of the hydroxy-terminated PBA in Table 3, WPU_2_ and WPU_3_ have the same meaning). Their particle size and particle size distributions are shown in Figure 7. Compared to WPU_2_, WPU_3_ and WPU_1_ displayed larger size and broader size distribution; this is probably attributable to the relatively long PBA chain in the case of WPU_1_ and higher content of the xylene unit in the case of WPU_3_ (see Table 3), because longer hydrophobic PBA chains benefit their assembly to form larger spherical particles, and more aromatic rings of the PBA chains will strengthen their interaction, facilitating their assembly.

The stability of aqueous PUDs is an important property for their application, and was characterized based on the size variation and dispersive state change of the aqueous PBA-based PUDs. The results listed in Table 4 reveal that the aqueous PBA-based PUDs were quite stable, as no precipitation was observed after centrifugation, and even after storage for two months.

**Figure 8:**
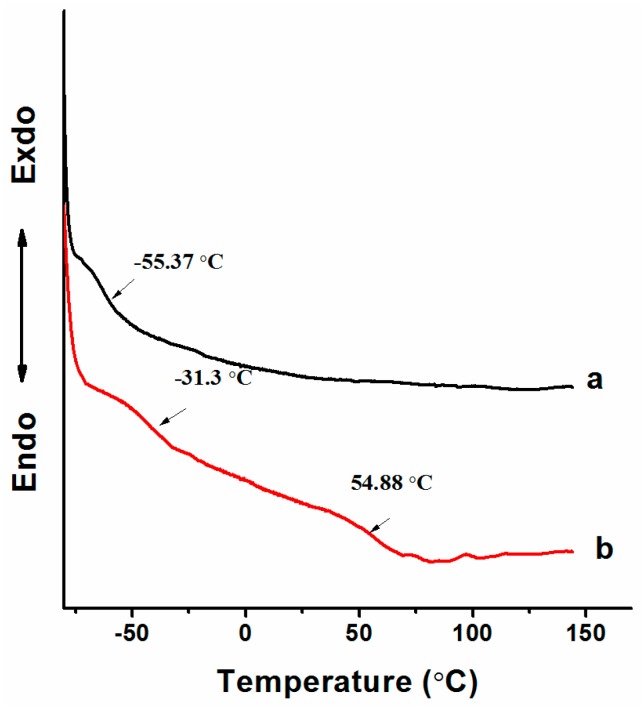
DSC thermograms of the hydroxyl-terminated PBA (a) and the PBA-based PUD (b).

As we mentioned in the Introduction, low compatibility between the polyurethane and the polyacrylate has limited the applications of waterborne polyurethane–polyacrylate hybrid emulsions [13,14,15]. Therefore, a DSC thermogram of the PBA-based PUD film was measured, and the result is shown in Figure 8. For comparison, the DSC thermogram of hydroxyl-terminated PBA is also shown in this figure. The glass transition (*T*_g_) of hydroxyl-terminated PBA at −55.4 °C can be clearly seen in Figure 8a. However, Figure 8b revealed two *T*_g_s: one at −31.3 °C, and another at 54.9 °C. The former is induced by the PBA soft segment, and the latter is due to the hard segment of the PBA-based PUDs, which indicates that the phase separation into soft and hard domains occurred during the formation of the PBA-based PUD film. The *T*_g_ of PBA in the PUD film was higher than the *T*_g_ of hydroxyl-terminated PBA probably because some hard chains penetrated into the soft domain. This indicates that the miscibility between PBA and hard chains was improved. 

## 4. Conclusions

A macromonomer, α,ω-dihydroxyl-functionalized PBA, has been successfully synthesized by degenerative iodine transfer polymerization of *n*-BA using 4,4′-azobis(4-cyano-1-pentanol) and diiodoxylene as initiator and chain transfer agent, respectively, and subsequently quantitative substitution reaction of iodine-terminated PBA with KCH_2_CH_2_OH. In the DITP of *n*-BA, the terminal groups of the resultant polymer chains come only from the initiator and the transfer agent, and three types of PBA chains are formed based on the end units. That is, initiator fragment (I)–PBA–transfer agent fragment (T), I–PBA–I, and T–PBA–T. When the AIBN was used as initiator in the DITP of *n*-BA with the DIX as transfer agent, the hydroxyl group at both ends of the PBA chains was relatively low; exactly one hydroxyl group at each end of every PBA chain could be obtained by using ACPO instead of AIBN as initiator in the DITP of *n*-BA. The low-molecular-weight PBA was a mixture of different PBA chains with different numbers of DIX units and different terminal units. Owing to irreversible termination between different types of growing chain radicals and different numbers of active centers in different PBA chains, the formed PBA possessed broad molecular weight distribution. Because the reactions in the DITP are complicated, the polymerization behavior is different from a typical living radical polymerization. The hydroxyl-terminated PBA can be efficiently applied in the preparation of aqueous PUDs which are stable, and the miscibility between hard segment and PBA chain has been improved.

## Figures and Tables

**Table 1:** Degenerative iodine transfer polymerization (DITP) condition and results of *n*-butyl acrylate (*n*-BA) using diiodoxylene (DIX) and 2,2′-azobis(isobutyronitrile) (AIBN) as transfer agent and initiator, respectively.

Exp. *	[*n*-BA]:[AIBN]:[CTAs]	Conv. (%)	*M*_n,GPC_ (g mol^−1^)	OH value (mg KOH/g)	Functionality of end-hydroxyl (%) ^a^
1	10:1:0.5	89	2310	15	42
2	10:1:1	87	2020	20	40
3	10:0.5:1	83	1910	25	54
4	10:0.24:1	78	1680	26	49
5	10:0.16:1	76	1540	28	48

* All of the reaction times were 7.5 h according the report [18]. ^a^ Calculated by ([*I*_CH2_/2]/([*I*_CH3_/6] + [*I*_CH2_/2]) × 100, where *I*_CH3_ is the integral value of methyl proton of the initiator fragment at 1.20–1.30 ppm, and *I*_CH2_ is the integral value of methylene protons of β-mercaptoethanol at 3.6–3.8 ppm.

**Table 2:** Structural formulas of the PBA produced in DITP of *n*-BA, depicted based on MALDI-TOF Mass Spectrum.

No.	Structural formula ^a^	(*m/z*)^observation^ (g/mol)	(*m/z*)^calc.^ (g/mol)
A	Na^+^HO–CPO–(*n*-BA)_n_–ME–OH	3158.4 (*n* = 23)	3159.2 (*n* = 23)
B	Na^+^HO–ME–(*n*-BA)_n_–xylene–(*n*-BA)_m_–ME–OH	3232.5 (*n*_2_ = 23)	3228.2 (*n*_2_ = 23)
C	Na^+^HO–CPO–(*n*-BA)_n_–xylene–(*n*-BA)_m_–xylene–(*n*-BA)_p_–xylene–(*n*-BA)_q_–ME–OH	3214.3 (*n*_4_ = 21)	3215.3 (*n*_3_ = 21)
D	Na^+^HO–CPO–(*n*-BA)_n_–xylene–(*n*-BA)_m_–xylene–(*n*-BA)_p_–CPO–OH	3271.6 (*n*_3_ = 23)	3274.4 (*n*_3_ = 23)
E	Na^+^HO–CPO–(*n*-BA)_n_–CPO–OH	3196.2 (*n* = 23)	3194.2 (*n* = 23)
F	Na^+^HO–ME–(*n*-BA)_n_–xylene–(*n*-BA)_m_–xylene–(*n*-BA)_p_–xylene–(*n*-BA)_q_–ME–OH	3181.1 (*n*_4_ = 21)	3180.3 (*n*_4_ = 21)
G	Na^+^HO–CPO–(*n*-BA)_n_–xylene–(*n*-BA)_m_–xylene–(*n*-BA)_p_–xylene–(*n*-BA)_q_–CPO–OH	3122.1 (*n*_4_ = 20)	3124.2 (*n*_4_ = 20)

^a^ CPO: 4-cyano-1-pentanol (HOCH_2_CH_2_CH_2_(CN)(CH_3_)C); ME: 2-mercaptoethanol (SCH_2_CH_2_OH); xylene: CH_2_C_6_H_5_CH_2_; *n*_2_ = n + m; *n*_3_ = n + m + p; *n*_4_ = n + m + p + q.

**Table 3:** Conditions and results obtained from DITP of *n*-BA at 60 °C for 24 h.

Exp.	[*n*-BA]: [ACPO]:[DIX] ^a^	Conv.^b^ (%)	*M*_n,NMR_^c^ (g mol^−1^)	*M*_n,GPC_^d^ (g mol^−1^)	*M*_n,MS_^e^ (g mol^−1^)	Functionality of end-hydroxyl (%) ^f^	OH_titr._ value (mg KOH/g)	OH_calc._^g^ value (mg KOH/g)
1	16:0.5:1	78	4409	4280	4090	76	29	27
2	16:0.25:1	75	3653	3480	3110	77	35	36
3	16:0.08:1	73	3545	3200	3230	72	31	35
4	16:0.04:1	71	3734	3160	3300	76	33	34

^a^ feed molar ratio; ^b^ Conv. = conversion, which was measured according to gravimetric method; ^c^ *M*_n,NMR_: obtained based on ^1^H NMR spectra of the resultant PBA; ^d^ *M*_n,GPC_: obtained by GPC method; ^e^ *M*_n,MS_: obtained by MALDI-TOF spectra; ^f^ Calculated by ([*I*_CH_]/([*I*_CH2_/2]) × 100, where *I*_CH2_ is the integral value of methylene protons linked with hydroxyl of the initiator fragment and β-mercaptoethanol at 3.62–3.93 ppm, and *I*_CH_ is the integral value of methine proton adjacent to sulfur at 2.67 ppm; ^g^ OH_calc._ = 56.1 × 2 × 1000/*M*_n,MS_, the empirical formula of OH value and *M*_n_.

**Table 4:** The stability and particle size of the aqueous PBA-based PUDs.

No.	OH ^a^ value	Dispersion state ^b^	Average particle size (nm)
1	29	Semi-transparent liquid	134
2	31	Semi-transparent liquid	100
3	33	Semi-transparent liquid	100

^a^ OH value of the hydroxyl-terminated PBA before preparation of the aqueous PBA-based PUDs; ^b^ No precipitation was observed after centrifugation.
